# Performance of a novel DiaRD-HCV RNA RTqPCR kit for quantification of HCV RNA in clinical samples

**DOI:** 10.1007/s10096-025-05179-5

**Published:** 2025-06-05

**Authors:** Ulker Cuhaci, Bengul Durmaz, Hande Toptan, Mehmet Koroglu, Derya Mutlu, Dilek Colak, Rıza Durmaz

**Affiliations:** 1 Faculty of Medicine, Department of Medical Microbiology, Yuksek İhtisas University, 06530 Ankara, Turkey; 2 Faculty of Medicine, Department of Medical Microbiology, Lokman Hekim University, 06530 Ankara, Turkey; 3 Faculty of Medicine, Department of Medical Microbiology, Sakarya University, 54050 Sakarya, Turkey; 4 Faculty of Medicine, Department of Medical Microbiology, Akdeniz University, 07070 Antalya, Turkey; 5 Faculty of Medicine, Department of Medical Microbiology, Yıldırım Beyazıt University, 06760 Ankara, Turkey

**Keywords:** HCV, QPCR, DiaRD HCV RTqPCR kit, Artus HCV QS-RGQ kit, NeuMoDx HCV quant test

## Abstract

**Purpose:**

Quantitative detection of hepatitis C virus (HCV) RNA is essential for diagnosis of chronic infection and monitoring the HCV levels in patients under treatment. In this study, we aimed to evaluate the performance characteristics and comparability of a novel DiaRD-HCV RT qPCR kit (Diagen Lts, Ankara, Turkey) with Artus HCV QS-RGQ kit and the NeuMoDx HCV Quant Test in clinical samples.

**Methods:**

A total of 240 plasma samples, 86 HCV RNA positive and 154 negative, were used for comparison. Intra-assay and inter-assay reproducibility tests of the novel kit were carried out on clinical samples having HCV RNA loads of 1.42 × 10^3^ IU/µl, 1.40 × 10^4^ IU/ml, and 5.10 × 10^5^ IU/ml. HCV genotypes 1b, 2a, 2b, 3a, 4, 5, and 6 were tested by the novel system.

**Results:**

DiaRD HCV RTqPCR kit demonstrated a sensitivity of 98.8%, a specificity of 100%, and an accuracy of 99.6%. Linear regression analysis demonstrated strong correlation (R^2^ = 0.915) on paired quantitative results. There was a good agreement between the novel kit and two comparator kits, with an overall mean difference of -0.12 log10 IU/ml obtained by Bland–Altman analysis. According to different viral loads, the intra-assay, inter-assay, and inter-load coefficients of variations (CVs) of the novel kit ranged from 5.31% to 11.73%, from 9.31% to 17.16%, and from 4.25% to 15.38% repeats. P-values were between 0.3 and 0.9, confirming no significant variations in the quantitative results of the reproducibility experiments. All genotypes tested were detected and quantitated with the mean differences between 0.01 and 0.62 log10 IU/ml. The primers and probe used in the novel kit did not result in any false-positive results with other pathogens.

**Conclusions:**

These results highlight the potential utility of the DiaRD HCV RTqPCR kit for quantitation and monitoring of HCV RNA in patients.

## Introduction

Hepatitis C virus (HCV) belongs to the genus *Hepacivirus* of the *Flaviviridae* family. It has a positive-stranded RNA with a genome size of approximately 9600 nucleotides [1]. The RNA genome is packaged within an icosahedral capsid, which is further encased by a lipid envelope [2]. Phylogenetic analyses have shown that there are eight major genotypes and 100 subtypes within HCV. Genotypes 1a, 1b, 2a, 2b, 2c, 3a, 4a, 5a, and 6a are responsible for most HCV infections in developed countries [3]. Genotypes 1 and 3 are the most common genotypes, responsible for more than 30% of all infections. Genotypes 2, 4, 5 and 6 are responsible for less than 10% of infections respectively. Genotype 7 has been identified in a few patients in Central Africa [1, 4].

Chronic HCV infection can lead to severe liver diseases, including steatosis, cirrhosis, and hepatocellular carcinoma (HCC) [1, 2]. The main mode of transmission of the hepatitis C virus is parenteral transmission through transfusion of blood and blood products, the use of contaminated needles, and intravenous drug use [5]. HCV infection is a significant global health problem throughout the world. According to the World Health Organization’s (WHO) 2024 Global Hepatitis Report, about 50 million people are living with hepatitis C worldwide and the estimated number of deaths due to viral hepatitis has risen from 1.1 million in 2019 to 1.3 million in 2022. Of these deaths, 17% are caused by hepatitis C [6].

One of the major challenges in the global elimination of HCV is inadequate diagnosis. The low diagnosis rate is a major barrier in resource-limited countries, primarily due to the cost of the molecular tools required to diagnose and monitor chronic HCV infection [1]. Quantitative real-time polymerase reaction (qPCR) systems are needed to determine chronic infection and to decide treatment in antibody positive patients and to confirm HCV eradication after treatment [1, 7–9]. The DiaRD-HCV RTqPCR kit is a qPCR assay that contains all the essential components for quantifying HCV RNA in the plasma of Hepatitis C patients. The kit includes primers designed to amplify a 105-base pair region within the 5'untranslated region (5'UTR) of the HCV genome, along with a fluorophore dye and a suppressor-labeled TaqMan probe to detect amplification. The kit has plasmid-derived internal control RNA with the same length and similar base content as the target gene, with a primer binding site identical to the target gene, but with a probe binding site which is unique to the target gene for ensuring the detection of potential PCR inhibitors. The kit also provides four quantitation standards, along with positive and negative controls for determination of HCV RNA load (https://www.diagen.com.tr/hepatitis-c-qualitative-rt-pcr-kit).

The aim of the study was to evaluate the clinical performance of a novel qPCR kit, DiaRD-HCV Hepatitis C RTqPCR kit by comparing it with two IVD-CE kits, which are Artus HCV QS-RGQ kit and NeuMoDx™ HCV Quant Test, and assessing the clinical utility of DiaRD-HCV in the diagnosis of Hepatitis C.

## Material and method

### Clinical samples

A total of 240 plasma samples, 86 of which were HCV RNA positive and 154 negative were retrospectively selected to evaluate the performance characteristics and comparability of a novel DiaRD-HCV RTqPCR (Diagen Lts, Ankara, Turkey). Two hundred and two samples, 48 positive and 154 negative for HCV RNA tested by Artus HCV QS-RGQ kit for routine detection and quantification of HCV RNA during 2023 were provided by the Medical Microbiology Laboratory of Sakarya University Education and Research Hospital in Sakarya/Turkey. Thirty-eight HCV RNA positive samples tested by NeuMoDx™ HCV Quant system during 2024 were obtained from Medical Microbiology Laboratory of Antalya University Education and Research Hospital in Antalya/Turkey. The plasma was separated within the first six hours after blood collection. Each plasma was aliquoted into two tubes, one tube of each sample was used for routine quantitation of HCV RNA by the comparator kits, the others were stored at −70 °C until processing with novel kit. Before testing, the patient information was anonymized. No specific information such as the patient’s name, surname, ID number, or phone number was used.

Ethical approval was obtained from Sakarya University, Medical Faculty Ethical Committee (Ethical Committee number: E-16214662–050.01.04–305620-153 and date of approval: 14.11.2023).

### RNA isolation

The RNA samples were extracted from plasma using the Diarex Viral DNA/RNA extraction kit protocol (Cat. No: VDR-8786, Diagen Inc, Ankara, Turkey https://www.diagen.com.tr). A total of 200 µl plasma was mixed with 25 µl of proteinase K, 12 µl of internal control, and 250 µl of lysis buffer including 2 µl carrier RNA. After spinning for 15 s, the tubes were incubated at 56ºC for 5 min. Then, 250 µl of absolute ethyl alcohol was added and vortexed for 15 s. The entire lysate was transferred to a spin column placed in a collection tube and centrifuged at 8000xg for 1 min. The spin column was placed back into the collection tube and 500 µL of wash buffer −1 (WBD-1) was added and centrifuged at 8000xg for 1 min. This step was repeated for wash buffer −2 (WBD-2). The spin column was placed in a new collection tube and 60 µL of RNA stabilizer was added and left for 2 min. Then it was centrifuged at 8000xg for 1 min. The genomic RNA collected in the collection tube was stored at + 4º or −20º for short-term study. For the Artus HCV QS-RGQ kit, HCV RNA isolation was performed on the QIAsymphony SP using a QIAsymphony DSP Virus/Pathogen Kit by strictly following kit’s instruction (file:///C:/Users/90532/AppData/Local/Temp/MicrosoftEdgeDownloads/9e0044c6-63ae-426 d-ba15-b435a7ae8742/HB-3178-002_HB_NeuMoDx_ 300300_HCV-Quant-Test-Strip_40600140_%20G_0723_ WW.pdf.). For the NeuMoDx HCV Quant assay, the automated NeuMoDx molecular system was used for extraction and purification of HCV RNA (file:///C:/Users/90532/AppData/Local/Temp/MicrosoftEdgeDownloads/9e0044c6-63ae-426 d-ba15-b435a7ae8742/HB-3178-002_HB_NeuMoDx_ 300300_HCV-Quant-Test-Strip_40600140_%20G_0723_ WW.pdf.).

## RTqPCR protocols

### The novel DiaRD-HCV RTqPCR kit

The novel DiaRD-HCV RTqPCR amplification mix, designed as a multiplex system, includes primers targeting a 105-base region within the 5’ untranslated region (5'UTR) of the HCV genome, a TaqMan probe, and internal control primers/probe. Four quantification standards are employed to determine the amount of HCV RNA. Reaction volume and amount of target RNA are 20 μl and 5 μl, respectively. An amplification program is constructed with a reverse- transcription step at 50 °C for 30 min and initial denaturation at 94 °C for 2 min, following 50 cycles including denaturation at 94 °C for 30 s, annealing at 55 °C for 50 s, and extension at 72 °C for 50 s. This novel quantitative kit has an analytical sensitivity of 20.8 IU/ml (0.0208 IU/μl), linearity range of < 15.7 IU/ml-1.57 × 106 IU/ml in the serail dilutions of a HCV RNA positive sample and < 13 IU/ml- > 1.3 × 1010 IU/ml in the serial dilutions of the synthetic plasmid in HCV negative plasma, and able to detect six main HCV genotypes (https://www.diagen.com.tr/hepatitis-c-qualitative-rt-pcr-kit).

### Artus HCV QS-RGQ kit

While RNA extraction and reaction setup were manually performed in the novel kit, in the Artus HCV QS-RGQ kit, these processes were performed by automated systems. The Artus HCV QS-RGQ kit, which is a ready-to-use system for the quantification of HCV RNA in plasma and serum, is used the QIAsymphony SP/AS instruments for sample preparation and assay setup. Total qPCR reaction volume and amount of target RNA are 50 μl and 20 μl, respectively. The kit has a reverse-transcription step at 50 °C for 30 min and initial denaturation at 95 °C for 15 min, following 50 cycles including denaturation at 95 °C for 30 s, annealing at 50 °C for 60 s, and extension at 72 °C for 30 s. Its analytical sensitivity and linearity range are 21 IU/ml and 35 −1.77 × 10^7^ IU/ml, respectively (file:///C:/Users/90532/AppData/Local/Temp/MicrosoftEdgeDownloads/55f5e462-50e9-4f03-84ed-80386c2ee432/EN-artus-HCV-QS-RGQ-Kit-Handbook.pdf).

### NeuMoDx HCV Quant assay

The NeuMoDx HCV Quant Assay is a CE-IVD approved, full automated, real-time reverse transcriptase PCR test for the amplification of conserved sequences in the 5’ untranslated region in the HCV genome and quantitation of RNA in plasma or serum. In manufacturer’s instructions, it is indicated that its required input volume is 700 µL, dynamic range of quantification for target RNA is 7.7–1.6 × 10^8^ IU/mL, and analytical sensitivity is 8 IU/ml (file:///C:/Users/90532/AppData/Local/Temp/MicrosoftEdgeDownloads/9e0044c6-63ae-426 d-ba15-b435a7ae8742/HB-3178-002_HB_NeuMoDx_300300_HCV-Quant-Test-Strip_40600140_%20G_0723_ WW.pdf.).

The Artus® HCV QS-RGQ kit (QIAGEN GmbH, Germany) and the NeuMoDx HCV Quant assay (QIAGEN GmbH, Germany) were used as the comparator kits. All samples were processed by the DiaRD-HCV RTqPCR kit and the comparator kits, adhering strictly to the respective kit protocols. The clinical efficacy of the DiaRD-HCV RTqPCR kit was assessed by comparing its results to those obtained from the comparator kits.

## Reproducibility of the novel kit

Intra-assay, inter-assay, and inter-load reproducibility tests were performed using clinical samples with HCV RNA loads of 1.42 × 10^3^ IU/µl, 5.10 × 10^5^ IU/ml, and 1.40 × 10^4^ IU/ml through eight RT-qPCR replicates conducted over three separate days. The precision data was calculated based on the Cycle threshold (Ct) values and HCV RNA loads.

### Analytical sensitivity/Limit of detection (LoD) analysis

Analytical sensitivity of DiaRD HCV RTqPCR kit was detected on RNA samples extracted from serial dilutions of a plasma sample with HCV RNA load of 1.3 × 10e5 IU/ml. Dilutions of 300 IU/ml, 130 IU/ml, 65 IU/ml, 32.5 IU/ml, 21.7 IU/ml, 16.25 IU/ml, 13 IU/ml were prepared in HCV negative plasma sample. RNA extraction from each dilution was performed using DiaRex RNA extraction kit (Cat. No: VDR-8786, Diagen Ltd, www.diagen.com.tr). RTqPCR assays were carried on RNA samples of each dilution in eight replicates on three separate days. The positivity obtained from each dilution was used in Probit analysis to estimate limit of detection value.

### Specificity of the primers and probe used in the DiaRD HCV RTqPCR kit

Both in silico and experimental analysis were performed to show specificity of the primers and probe with six major HCV genotypes and to rule out cross-reactivity with other pathogens. RTqPCR studies were done on the Hepatitis B virus, Hepatitis D virus, Human immunodeficiency virus 1, Epstein-Barr virus, Cytomegalovirus, Human Herpesvirus types 1, Parvovirus B19, BK Virus, and *Escherichia coli* positive samples*.*

## Detection of HCV genotype

The HCV genotype panel (HCV RNA Genotype AccuTrak Qualification Panel, Seracare) was also used for the evaluation of the DiaRD HCV RT-qPCR assay. This panel is composed by seven samples of genotypes 1–6 (1b, 2a, 2b, 3a, 4, 5, and 6) of HCV with titers ranging from 1.62E + 04 to 1.85E + 05 IU/ml (as determined by Roche AmpliPrep/COBAS TaqMan HCV or Roche cobas 6800) (https://www.seracare.com/HCV-RNA-Genotype-1a-Positive-Plasma-SC0310-0042/).

### Statistical analysis

SPSS Software Version 17 was used for statistical analysis. Sensitivity, specificity and overall agreement were calculated to evaluate diagnostic performance of the novel kit. A regression analysis of the log10-transformed values was performed to show the correlation between HCV RNA viral load values obtained by the DiaRD HCV RTqPCR assay and the comparator kit. In addition, the Bland–Altman plots were used for the analysis of agreement between quantitative results of both kits. Ct values and HCV RNA loads of the samples used in reproducibility experiments were used to calculate standard deviation of the means, variation, and coefficient of variation (CV) values. CV values were used to evaluate performance of quantitative kits in terms of precision.

## Results

Eighty-five of 86 plasma samples identified as HCV RNA positive by comparator kits were also positive with the novel DiaRD-HCV RTqPCR kit. A sample having RNA load of 66 IU/ml by the comparator NeuMoDX HCV kit was found as negative by the novel kit. All 154 HCV negative samples also showed negative qPCR result with the novel kit. The sensitivity, specificity, and overall agreement of the novel kit was determined to be 98.8%, > 99.9%, and 99.6%, respectively. The kit showed a positive predictive value of 100%, and a negative predictive value of 99.6% (Table 1).

**Table 1:** Comparison of DiaRD HCV RTqPCR kit with the Comparator kits*

	Comparator kits		
DiaRD HCV RTqPCR kit	Positive	Negative	Total
Positive	85	0	85
Negative	1**	154	155
Total	86	154	240
Sensitivity (%)	98.8	−	−
Specificity (%)	−	100	
Overall agreement (%)	−	−	99.6
Positive predictive value (%)	100	−	−
Negative predictive value (%)	−	99.6	−

^*^ Comparator kits are the Artus HCV QS-RGQ kit and NeuMoDX HCV kit. **One sample, which had RNA load of 6.6 × 10^1^ IU/ml with NeuMoDX HCV kit was negative with the novel kit

The median viral load values of the novel kit, the Artus HCV QS-RGQ kit, and the NeuMoDX HCV kit were as 1.89 × 10^6^ IU/ml (range: 2.3 × 10^1^—1.35 × 10^7^), 6.43 × 10^5^ (range: 2.50 × 10^1^–8.85 × 10^6^), and 1.89 × 10^6^ (range:69–1.10 × 10^7^), respectively. When the results were analyzed in log10 IU/ml, the median viral load value was 5.28 log10 IU/ml (range: 1.36 to 7.13) for the novel kit, 4.86 log10 IU/ml (range: 1.4 to 6.77) for the Artus HCV QS-RGQ kit and 5.63 (range:1.84 to 7.04) for the NeuMoDX HCV kit. Among the 85 samples having positive results with the novel and comparator kits, 74 (86.05%) had a measurement difference ≤  ± 0.5 log10 IU/ml, and 12 (13.95%) had a difference of >  ± 0.5 log10 (range = 0.59 to 1.22 log10). According to comparator kits, these 12 samples had RNA concentrations between 2.5 × 10^1^ and 1.3 × 10^6^ IU/ml (mean = 7.76 × 10^5^ IU/ml). In these 12 samples, one had an RNA load of 2.50 × 10^1^ IU/ml, two had RNA load of 2.89 × 10^3^ and 5.19 × 10^3^ IU/ml, three had RNA load ranging from 2.40 × 10^4^ to 4.90 × 10^4^ IU/ml, five had RNA load ranging from 1.28 × 10^5^ to 4.90 × 10^5^ and one had RNA level of 1.30 × 10^6^ IU/ml. In a sample with HCV RNA load of 5.20 × 10^6^ IU/ml by the novel kit, the comparator Artus RGS HCV kit detected lower RNA load (1.30 × 10^6^ IU/ml). The novel kit detected higher RNA loads in nine of the 12 samples having RNA showing difference of >  ± 0.5 log10 IU/ml. There was one sample having significant difference of ≥ 1 log10 IU/ml (Table 2).

**Table 2:** Comparison of HCV RNA loads detected by the DiaRD HCV RTqPCR kit and comparator kits (Difference > 5 log10 indicated as bold)

Sample no	Artus HCV QS-RGQ kit results (IU/ml)	DiaRD HCV RTqPCR results (IU/ml)	Mean between two kits (IU/ml))	SD between two kits (IU/ml))	Artus HCV QS-RGQ kit results (log10)	DiaRD HCV RTqPCR results (log 10)	Mean between two kits (Log10)	SD between two kits (log10)	Difference between Log10
1	7.3 × 10^5^	1.57 × 10⁶	1.15 × 10⁶	4.20 × 10^5^	5.86	6.20	6.03	0.17	−0.33
2	5.4 × 10^5^	9.84 × 10^5^	7.62 × 10^5^	2.22 × 10^5^	5.73	5.99	5.86	0.13	−0.26
3	5.85 × 10⁶	1.3 × 10⁷	9.43 × 10⁶	3.58 × 10⁶	6.77	7.11	6.94	0.17	−0.35
4	7.13 × 10^4^	1.57 × 10^5^	1.14 × 10^5^	4.29 × 10^4^	4.85	5.20	5.02	0.17	−0.34
5	3.17 × 10⁶	8.65 × 10⁶	5.91 × 10⁶	2.74 × 10⁶	6.50	6.94	6.72	0.22	−0.44
6	7.4 × 10^5^	1.3 × 10⁶	1.02 × 10⁶	2.80 × 10^5^	5.87	6.11	5.99	0.12	−0.24
7	6.07 × 10^3^	1.57 × 10^4^	1.09 × 10^4^	4.82 × 10^3^	3.78	4.20	3.99	0.21	−0.41
8	2.4 × 10^5^	4.92 × 10^5^	3.66 × 10^5^	1.26 × 10^5^	5.38	5.69	5.54	0.16	−0.31
9	4.4 × 10^4^	1.3 × 10^5^	8.70 × 10^4^	4.30 × 10^4^	4.64	5.11	4.88	0.24	−0.47
10	5.64 × 10^2^	1.57 × 10^3^	1.07 × 10^3^	5.03 × 10^2^	2.75	3.20	2.97	0.22	−0.44
11	3.91 × 10⁶	9.9 × 10⁶	6.91 × 10⁶	3.00 × 10⁶	6.59	7.00	6.79	0.20	−0.40
12	2.89 × 10^3^	1.3 × 10^4^	7.95 × 10^3^	5.06 × 10^3^	3.46	4.11	3.79	0.33	**−0.65**
13	2.5 × 10^1^	1.57 × 10^2^	9.10 × 10^1^	6.60 × 10^1^	1.40	2.20	1.80	0.40	**−0.80**
14	1.09 × 10^4^	2.98 × 10^4^	2.04 × 10^4^	9.45 × 10^3^	4.04	4.47	4.26	0.22	−0.44
15	5.67 × 10^4^	1.3 × 10^5^	9.34 × 10^4^	3.67 × 10^4^	4.75	5.11	4.93	0.18	−0.36
16	1.12 × 10^3^	1.63 × 10^3^	1.38 × 10^3^	2.55 × 10^2^	3.05	3.21	3.13	0.08	−0.16
17	5.75 × 10^4^	6.1 × 10^4^	5.93 × 10^4^	1.75 × 10^3^	4.76	4.79	4.77	0.01	−0.03
18	2.9 × 10^4^	3.85 × 10^4^	3.38 × 10^4^	4.75 × 10^3^	4.46	4.59	4.52	0.06	−0.12
19	1.23 × 10^4^	9.97 × 10^3^	1.11 × 10^4^	1.17 × 10^3^	4.09	4.00	4.04	0.05	0.09
20	1.3 × 10⁶	5.2 × 10⁶	3.25 × 10⁶	1.95 × 10⁶	6.11	6.72	6.41	0.30	**−0.60**
21	1.4 × 10^5^	1.00 × 10⁶	5.70 × 10^5^	4.30 × 10^5^	5.15	6.00	5.57	0.43	**−0.85**
22	4.7 × 10^5^	1.35 × 10⁶	9.10 × 10^5^	4.40 × 10^5^	5.67	6.13	5.90	0.23	−0.46
23	3.62 × 10⁶	4.91 × 10⁶	4.26 × 10⁶	6.47 × 10^5^	6.56	6.69	6.62	0.07	−0.13
24	1.37 × 10⁶	1.05 × 10⁶	1.21 × 10⁶	1.64 × 10^5^	6.14	6.02	6.08	0.06	0.12
25	1.05 × 10⁶	3.2 × 10⁶	2.13 × 10⁶	1.07 × 10⁶	6.02	6.50	6.26	0.24	−0.48
26	2.01 × 10⁶	2.96 × 10⁶	2.49 × 10⁶	4.73 × 10^5^	6.30	6.47	6.39	0.08	−0.17
27	7.04 × 10^5^	5.09 × 10^5^	6.07 × 10^5^	9.75 × 10^4^	5.85	5.71	5.78	0.07	0.14
28	1.69 × 10^4^	5.33 × 10^4^	3.51 × 10^4^	1.82 × 10^4^	4.23	4.73	4.48	0.25	−0.50
29	4.08 × 10^4^	1.01 × 10^4^	2.54 × 10^4^	1.54 × 10^4^	4.61	4.00	4.31	0.30	**0.61**
30	3.56 × 10^3^	2.75 × 10^3^	3.15 × 10^3^	4.08 × 10^2^	3.55	3.44	3.50	0.06	0.11
31	9.18 × 10^3^	4.68 × 10^3^	6.93 × 10^3^	2.25 × 10^3^	3.96	3.67	3.82	0.15	0.29
32	2.45 × 10^4^	8.57 × 10^3^	1.65 × 10^4^	7.95 × 10^3^	4.39	3.93	4.16	0.23	0.46
33	1.68 × 10^5^	1.76 × 10^5^	1.72 × 10^5^	4.05 × 10^3^	5.23	5.25	5.24	0.01	−0.02
34	1.28 × 10^5^	2.39 × 10^4^	7.62 × 10^4^	5.23 × 10^4^	5.11	4.38	4.74	0.37	**0.73**
35	1.37 × 10⁶	1.06 × 10⁶	1.22 × 10⁶	1.54 × 10^5^	6.14	6.03	6.08	0.06	0.11
36	1.65 × 10^4^3	1.95 × 10^4^	1.80 × 10^4^	1.47 × 10^3^	4.22	4.29	4.25	0.04	−0.07
37	5.55 × 10^5^	4.91 × 10^5^	5.23 × 10^5^	3.22 × 10^4^	5.74	5.69	5.72	0.03	0.05
38	1.7 × 10^5^	1.05 × 10^5^	1.37 × 10^5^	3.28 × 10^4^	5.23	5.02	5.13	0.11	0.21
39	2.11 × 10^5^	3.2 × 10^5^	2.66 × 10^5^	5.42 × 10^4^	5.33	5.50	5.41	0.09	−0.18
40	3.62 × 10^5^	2.96 × 10^5^	3.29 × 10^5^	3.31 × 10^4^	5.56	5.47	5.52	0.04	0.09
41	1.74 × 10^5^	8.09 × 10^4^	1.12 × 10^5^	5.13 × 10^4^	5.24	4.91	4.97	0.27	0.33
42	3.26 × 10^3^	5.33 × 10^3^	4.29 × 10^3^	1.03 × 10^3^	3.51	3.73	3.62	0.11	−0.21
43	5.19 × 10^3^	1.01 × 10^3^	3.10 × 10^3^	2.09 × 10^3^	3.72	3.00	3.36	0.36	**0.71**
44	2.62 × 10^2^	2.75 × 10^2^	2.68 × 10^2^	6.25 × 10⁰	2.42	2.44	2.43	0.01	−0.02
45	1.3 × 10⁶	1.18 × 10⁶	1.24 × 10⁶	6.00 × 10^4^	6.11	6.07	6.09	0.02	0.04
46	1.3 × 10^5^	1.41 × 10^5^	1.36 × 10^5^	5.50 × 10^3^	5.11	5.15	5.13	0.02	−0.04
47	1.3 × 10^4^	1.49 × 10^4^	1.40 × 10^4^	9.50 × 10^2^	4.11	4.17	4.14	0.03	−0.06
48	1.3 × 10^3^	1.16 × 10^3^	1.23 × 10^3^	7.00 × 10^1^	3.11	3.06	3.09	0.02	0.05
	NeuMoDx HCV Quant Test results (IU/ml)	DiaRD HCV RTqPCR results (IU/ml)	Mean between two kits (IU/ml))	SD between two kits (IU/ml))	NeuMoDx HCV Quant Test results (log10)	DiaRD HCV RTqPCR results (log 10)	Mean between two kits (Log10)	SD between two kits (log10)	Difference between Log10
49	5.1 × 10⁶	6.06 × 10⁶	5.58 × 10⁶	4.82 × 10^5^	6.71	6.78	6.75	0.04	−0.08
50	7.2 × 10^5^	2.51 × 10^5^	4.85 × 10^5^	2.35 × 10^5^	5.86	5.40	5.63	0.23	0.46
51	5.6 × 10⁶	1.35 × 10⁷	9.54 × 10⁶	3.94 × 10⁶	6.75	7.13	6.94	0.19	−0.38
52	1.1 × 10⁷	1.22 × 10⁷	1.16 × 10⁷	6.20 × 10^5^	7.04	7.09	7.06	0.02	−0.05
53	69	30	4.95 × 10^1^	1.95 × 10^1^	1.84	1.48	1.66	0.18	0.36
54	2.8 × 10^4^	9.89 × 10^3^	1.89 × 10^4^	9.06 × 10^3^	4.45	4.00	4.22	0.23	0.45
55	6.8 × 10^4^	1.3 × 10^5^	9.91 × 10^4^	3.11 × 10^4^	4.83	5.11	4.97	0.14	−0.28
56	4.24 × 10^5^	3.21 × 10^5^	3.72 × 10^5^	5.16 × 10^4^	5.63	5.51	5.57	0.06	0.12
57	3.5 × 10^5^59	2.6 × 10^5^	3.05 × 10^5^	4.48 × 10^4^	5.54	5.42	5.48	0.06	0.13
58	2.4 × 10^5^	2.09 × 10⁶	1.17 × 10⁶	9.27 × 10^5^	5.38	6.32	5.85	0.47	**−0.94**
59	4.9 × 10^5^	3.77 × 10⁶	2.13 × 10⁶	1.64 × 10⁶	5.69	6.58	6.13	0.44	**−0.89**
60	3.05 × 10⁶	6.28 × 10⁶	4.67 × 10⁶	1.62 × 10⁶	6.48	6.80	6.64	0.16	−0.31
61	6.68 × 10⁶	1.14 × 10⁷	9.04 × 10⁶	2.36 × 10⁶	6.82	7.06	6.94	0.12	−0.23
62	1.25 × 10⁶	2.39 × 10⁶	1.82 × 10⁶	5.72 × 10^5^	6.10	6.38	6.24	0.14	−0.28
63	1.64 × 10⁶	2.29 × 10⁶	1.96 × 10⁶	3.24 × 10^5^	6.21	6.36	6.29	0.07	−0.14
64	1.41 × 10⁶	8.13 × 10^5^	1.11 × 10⁶	2.98 × 10^5^	6.15	5.91	6.03	0.12	0.24
65	5.24 × 10^5^6	6.34 × 10^5^	5.79 × 10^5^	5.52 × 10^4^	5.72	5.80	5.76	0.04	−0.08
66	3.81 × 10⁶	5.4 × 10⁶	4.60 × 10⁶	7.94 × 10^5^	6.58	6.73	6.66	0.08	−0.15
67	2.75 × 10⁶	3.15 × 10⁶	2.95 × 10⁶	2.02 × 10^5^	6.44	6.50	6.47	0.03	−0.06
68	8.3 × 10⁶	9.52 × 10⁶	8.91 × 10⁶	6.09 × 10^5^	6.92	6.98	6.95	0.03	−0.06
69	2.74 × 10^4^	1.76 × 10^4^	2.25 × 10^4^	4.91 × 10^3^	4.44	4.25	4.34	0.10	0.19
70	2.1 × 10^5^	3.51 × 10⁶	1.86 × 10⁶	1.65 × 10⁶	5.32	6.55	5.93	0.61	**−1.22**
71	2.72 × 10⁶	6.14 × 10⁶	4.43 × 10⁶	1.71 × 10⁶	6.43	6.79	6.61	0.18	−0.35
72	5.1 × 10^5^	1.34 × 10⁶	9.27 × 10^5^	4.17 × 10^5^	5.71	6.13	5.92	0.21	−0.42
73	7.2 × 10^4^	1.87 × 10^5^	1.29 × 10^5^	5.74 × 10^4^	4.86	5.27	5.06	0.21	−0.41
74	5.6 × 10^5^	1.35 × 10⁶	9.54 × 10^5^	3.94 × 10^5^	5.75	6.13	5.94	0.19	−0.38
75	1.1 × 10⁶	2.42 × 10⁶	1.76 × 10⁶	6.60 × 10^5^	6.04	6.38	6.21	0.17	−0.34
76	2.8 × 10^3^	7.89 × 10^3^	5.35 × 10^3^	2.55 × 10^3^	3.45	3.90	3.67	0.22	−0.45
77	4.24 × 10^4^	2.3 × 10^4^	3.27 × 10^4^	9.70 × 10^3^	4.63	4.36	4.49	0.13	0.27
78	3.5 × 10^4^	1.34 × 10^4^	2.42 × 10^4^	1.08 × 10^4^	4.54	4.13	4.34	0.21	0.42
79	2.4 × 10^4^	1.76 × 10^5^	1.00 × 10^5^	7.60 × 10^4^	4.38	5.25	4.81	0.43	**−0.87**
80	4.9 × 10^4^	1.92 × 10^5^	1.20 × 10^5^	7.14 × 10^4^	4.69	5.28	4.99	0.30	**−0.59**
81	3.05 × 10^5^	2.21 × 10^5^	2.63 × 10^5^	4.20 × 10^4^	5.48	5.34	5.41	0.07	0.14
82	6.68 × 10⁶	3.89 × 10⁶	5.29 × 10⁶	1.39 × 10⁶	6.82	6.59	6.71	0.12	0.23
83	1.25 × 10⁶	8.52 × 10^5^	1.05 × 10⁶	1.99 × 10^5^	6.10	5.93	6.01	0.08	0.17
84	1.64 × 10⁶	7.24 × 10^5^	1.18 × 10⁶	4.58 × 10^5^	6.21	5.86	6.04	0.18	0.35
85	1.41 × 10⁶	5.96 × 10^5^	1.00 × 10⁶	4.07 × 10^5^	6.15	5.78	5.96	0.19	0.37

As shown in Table 3, there was high coefficients of variation (CV) between the quantitative results of the novel and comparator kits in samples having RNA loads < 4 log10 IU/ml.

**Table 3:** Comparison of the results of DiaRD HCV RTqPCR kit and comparator kits by means of SD and % CV as per log10 values

Log10 values	Comparator kits				DiaRD HCV RTqPCR kit			
	n	Mean	SD	CV %	n	Mean	SD	CV %
< 4	14	2.99	0.79	26.4	12	2.97	0.86	28.9
4–5	20	4.50	0.25	5.55	16	4.32	0.25	5.78
5–6	25	5.53	0.25	4.52	25	5.47	0.29	5.30
> 6	27	6.42	0.30	4.67	32	6.54	0.35	5.35

**Fig. 1:**
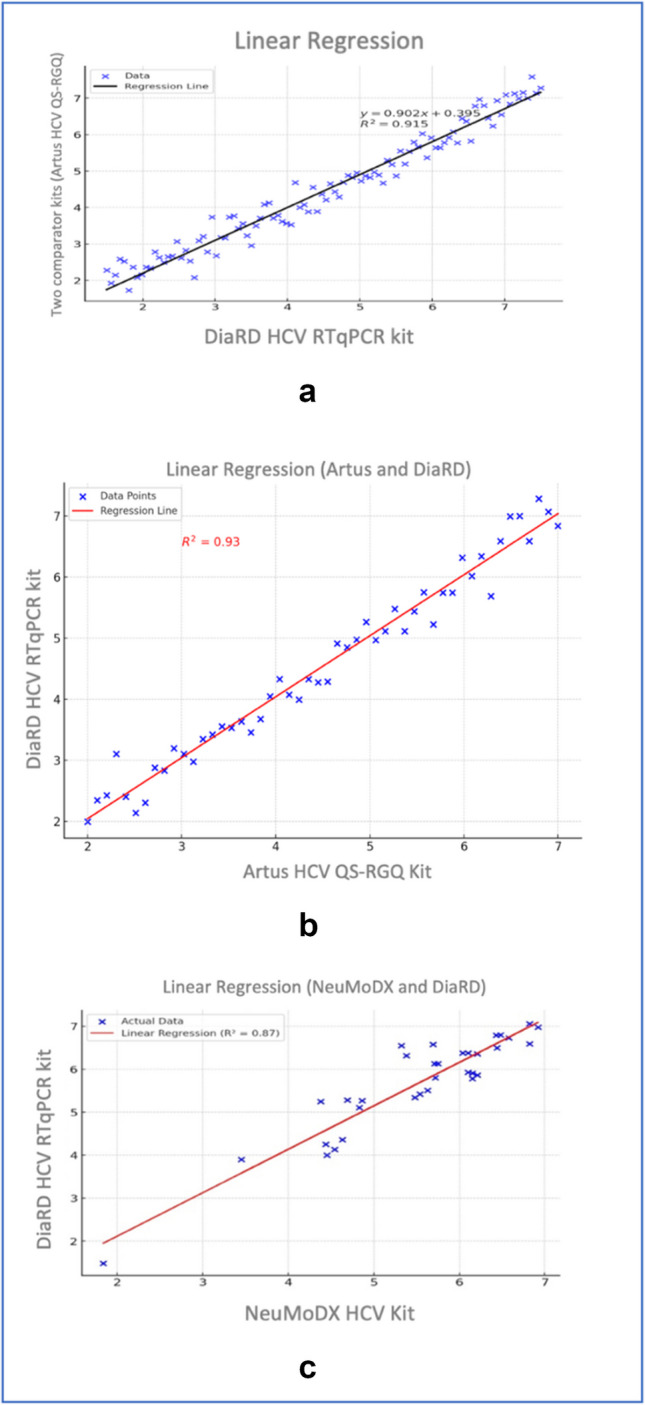
Linear regression analysis between quantitative results of DiaRD HCV RTqPCR kit and two comparator kits (1a), DiaRD HCV RTqPCR kit and Artus HCV QS-RGQ kit (1b), and DiaRD HCV RTqPCR kit and the NeuMoDX HCV kit (1c)

Linear regression analyses of 85 samples, which were within the linear dynamic ranges of the novel kit and comparator kits, demonstrated that the quantitative results of novel kit were highly corelated with those of the comparator kits (R ^2^ ≥ 0.915) (Fig. 1a). When the novel kit was compared with the Artus HCV QS-RGQ kit and the NeuMoDX HCV kit separately, R^2^ values were estimated as 0.93 (1b) and 0.87 (1c), respectively.

**Fig. 2:**
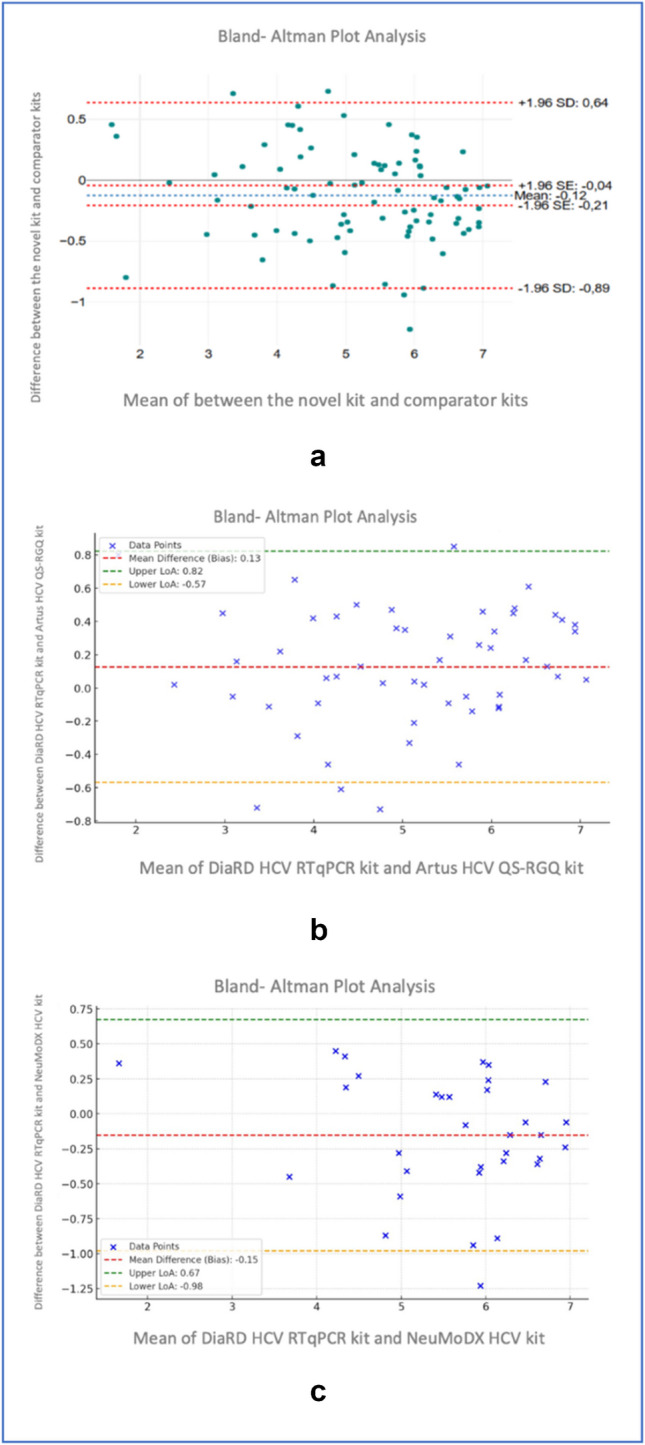
Bland–Altman analysis showing the mean log difference, upper limit of agreement, and lower limit of agreement between the novel kit and comparator kits (2a), the novel kit and Artus HCV QS-RGQ kit, and the novel kit and the NeuMoDX HCV kit (2c)

The difference between the HCV viral load log10 values obtained from 85 samples by the novel kit and comparator kits was plotted against the average of the log10 results of the kits tested. The differences between the novel and comparator tests were within the upper (+ 1.96 SD:0.64) and lower (−1.96 SD: −0.89) limits of agreement for 81 of the 85 samples (95% confidence interval, CI). The lowest difference between the comparator kits and the novel kit values was −1.22 log10, the highest difference was 0.73 log10, and the mean difference was −0.12 [standard deviation (SD) = 0.39] with a 95% CI (Fig. 2a). When Bland–Altman analysis was performed between the novel kit and the Artus HCV QS-RGQ kit (2b), and the novel kit and the NeuMoDX HCV kit (2c) separately, the mean difference was found to be 0.12 and 0.15, respectively.

Reproducibility of the assays were evaluated by intra-assay, inter-assay, and inter-load variations using three different HCV RNA concentrations. Each sample was run eight times in three days. According to Ct values, the intra-assay, inter-assay, and inter-load coefficients of variation (CVs) ranged from 0.27% to 0.70%, from 0.48% to 0.64%, and from 0.23% to 1.93%, respectively. Total variation was between 1.19% and 1.81%. P-values (0.2 to 0.6) showed no significant differences in the results of the reproducibility studies (Table 4). According to HCV RNA loads, the CV values were found between 5.31% and 11.73% for intra-assay, between 9.31% and 17.16% for inter-assay, and between 4.25% and 15.38% for inter-load repeats. P-values ranged from 0.3 to 0.9, confirming no significant variations in the quantitative results of the reproducibility experiments (Table 5).

**Table 4:** Reproducibility of the DiaRD HCV RTqPCR kit according to Ct values

HCV RNA 5,10E + 05 IU/ml	Mean (Ct values)	Standard Deviation	CV (%)	*P*-Value
Intra-assay	29.49	0.21	0.70	0.2
Inter-assay	29.01	0.18	0.62	
Inter-load	30.90	0.13	0.44	
Total variation	29.80	0.84	2.81	
HCV RNA 1,40E + 04 IU/ml				0.2
Intra-assay	32.27	0.09	0.27	
Inter-assay	32.34	0.16	0.48	
Inter-load	34.13	0.08	0.23	
Total variation	32.91	0.88	2.69	
HCV RNA 1,42E + 03 IU/ml				0.6
Intra-assay	33.30	0.17	0.50	
Inter-assay	33.24	0.21	0.64	
Inter-load	33.09	0.64	1.93	
Total variation	33.21	0.39	1.19

**Table 5:** Reproducibility of the DiaRD HCV RTqPCR kit according to HCV RNA loads

HCV RNA 5,10E + 05 IU/ml	Mean (RNA loads)	Standard Deviation	CV (%)	*P*-Value
Intra-assay	7.44 × 10^4^	8.54 × 10^3^	11.73	0.9
Inter-assay	1.0 × 10^5^	1.43 × 10^4^	11.63	
Inter-load	9.87 × 10^4^	5.69 × 10^2^	7.87	
Total variation	9.10 × 10^4^	1.32 × 10^4^	15.63	
HCV RNA 1,40E + 04 IU/ml				0.3
Intra-assay	1.32 × 10^4^	6.20 × 10^1^	5.31	
Inter-assay	1.34 × 10^4^	5.92 × 10^3^	9.31	
Inter-load	1.33 × 10^4^	1.13 × 10^2^	4.25	
Total variation	9.41 × 10^3^	5.63 × 10^3^	65.15	
HCV RNA 1,42E + 03 IU/ml				0.8
Intra-assay	1.42 × 10^3^	1.49 × 10^2^	8.52	
Inter-assay	1.34 × 10^3^	2.30 × 10^2^	17.16	
Inter-load	1.34 × 10^3^	2.06 × 10^2^	15.38	
Total variation	1.38 × 10^3^	1.70 × 10^2^	12.35	

**Fig. 3:**
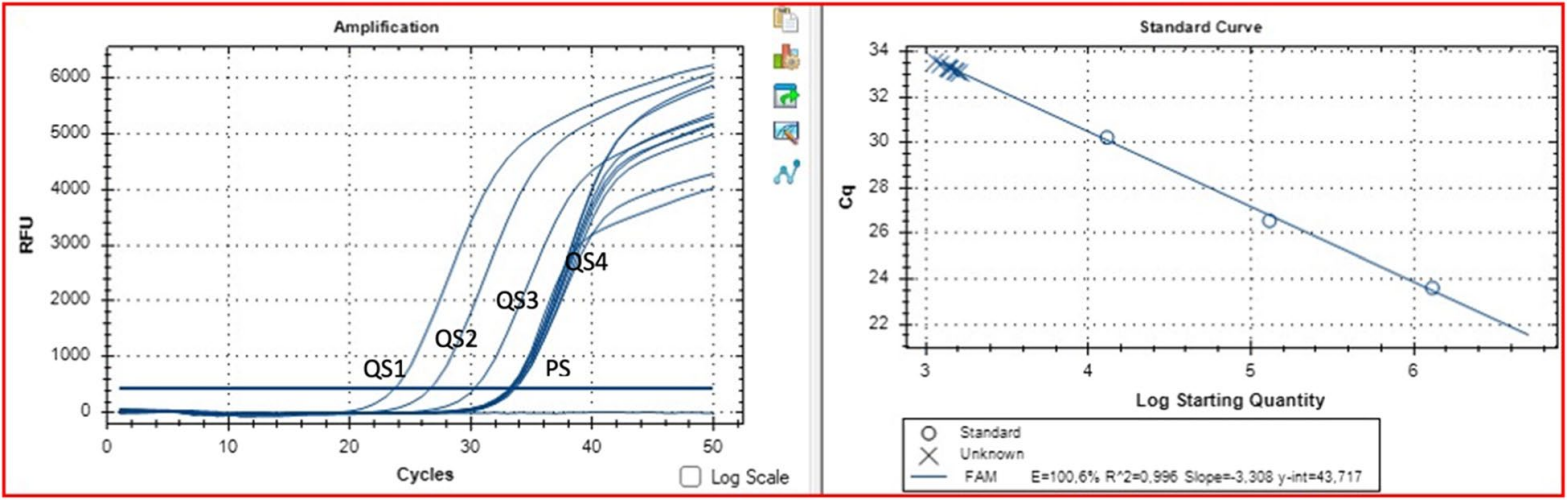
A representative example of DiaRD HCV RTqPCR reproducibility study. QS 1: Quantitation standard 1(4 × 10^7^ IU/ml), QS 2 (4 × 10^6^ IU/ml), QS 3 (4 × 10^5^ IU/ml), QS 4 (4 × 10^4^ IU/ml). PS: Patient sample (1.42 × 10^3^ IU/ml)

An example of relative fluorescent signal (RFU), PCR efficiency, and R^2^ values obtained by intra-assay repeats of the sample having RNA load of 1.42E + 03 IU/ml was presented in Fig. 3.

To determine whether the novel kit covers the main HCV genotypes (genotypes 1–6), members of the SeraCare HCV RNA Genotype AccuTrak™ Qualification Panel (genotypes 1b, 2a, 2b, 3a, 4, 5, and 6) in serum were tested on the system, and the measured log10 titers were compared to the respective log10 assigned values. All genotypes were detected with mean differences ranging from 0.01 to 0.62 log10 IU/mL between the measured and assigned values (Table 6).

**Table 6:** The results of genotypes of DiaRD HCV RT-qPCR kit in reference samples

HCV genotype (Batch no)	DiaRD HCV RTqPCR results		SeraCare values		
	IU/ml	Log10	IU/ml	Log10	Difference between log 10
1a (9,160,834)	1.33E + 06	6.12	9.97E + 05		0.12
1b (10,632,137)	1.08E + 06	6.03	1.57E + 06	6.19	−0.16
2 (10,632,145)	4.24E + 06	6.63	3.02E + 06	6.48	0.15
3a (9,145,669)	2.74E + 04	4.44	2.96E + 04	4.47	−0.03
4 (10,693,272)	1.01E + 04	4.00	4.16E + 04	4.62	**−0.62**
5 (10,408,500)	3.00E + 05	5.48	3.05E + 05	5.48	0.01
6 (10,693,273)	5.20E + 08	8.72	2.90 E + 08	8.46	0.26

**Fig. 4:**
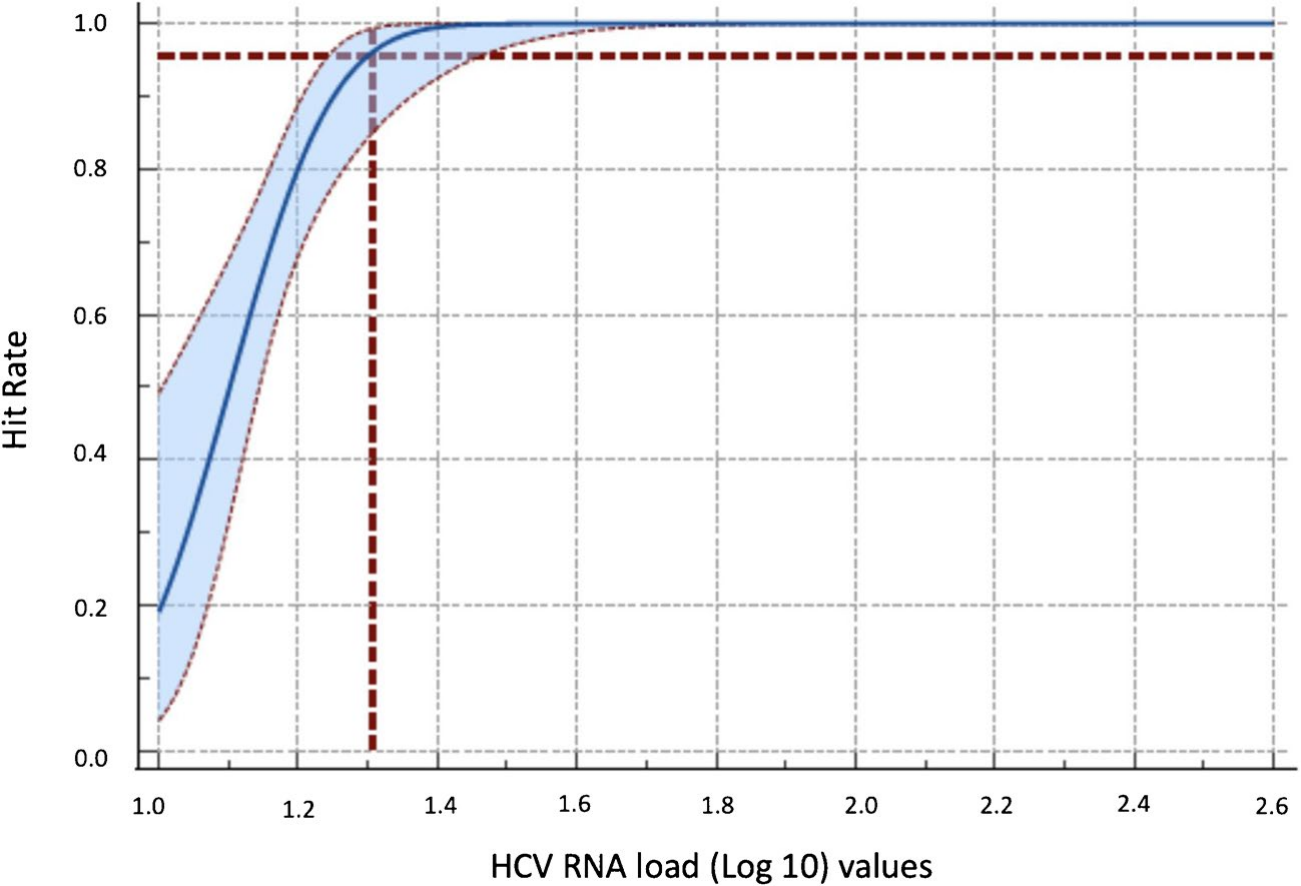
Probit analysis shows the analytical sensitivity of the DiaRD HCV RTqPCR kit. Digits in the horizontal lines belonged to the log10 values of the HCV RNA loads (log 10 1.1 = 13 IU/ml, log10 1.2 = 16.25 13 IU/ml, log10 1.3 = 21.7 IU/ml, log10 1.5 = 32.5 IU/ml, log10 1.8 = 65 IU/ml, log10 2.1 = 130 IU/ml, log10 2.47 = 300 IU/ml) tested in analytical sensitivity studies

According to results of probit analysis, analytical sensitivity/LoD value of DiaRD HCV RTqPCR kit was detected as 20.8 IU/ml of HCV RNA (Fig. 4). This HCV RNA load (20.8 IU/ml) can be consistently detected with a 95% certainty.

In silico analyses showed that primers and probe used in DiaRD HCV RTqPCR kit were highly specific having sequence homology of ≥ 88% with HCV genotype 1 A, IB, 2, 3, 4, 5, and 6 genomes. The rate of homology between primers and HCV genotypes ranged from 88 to 100%. This rate was 96 to 100% between probe and HCV genotypes. Whilst only less than half bases in each primers/probe showed homology of ≤ 50% with the genome of the other pathogens tested. RTqPCR studies with these primers and probe yielded positive results in all major HCV genotypes, without any false-positive reaction with other pathogens (Table 7).

**Table 7:** Cross-reactivity studies with primers/probes used in DiaRD HCV RTqPCR kit

	DiaRD HCV RTqPCR kit	
Tested pathogens	Ct value*	Result
Epstein Barr virus (EBV)	NA	Negative
Cytomegalovirus (CMV)	NA	Negative
Human Herpesvirus 1	NA	Negative
Parvovirus B19	NA	Negative
BK Virus	NA	Negative
Hepatitis B virus	NA	Negative
Hepatitis D virus	NA	Negative
Human immunodeficiency virus (HIV)	NA	Negative
*Escherichia coli*	NA	Negative
HCV genotypes		
1 A	25.05	Positive
1B	25.39	Positive
2	23.19	Positive
3	27.58	Positive
4	25.48	Positive
5	35.00	Positive
6	26.55	Positive

^*^NA: Not available

## Discussion

The quantitative detection of HCV RNA has been pivotal for initiating treatment and monitoring RNA levels in patients undergoing therapy. Numerous commercial qPCR kits are available for the detection and quantification of HCV RNA [8–13]. Among these assays, selecting the most accurate and reliable kits is essential for ensuring effective clinical management. This study evaluated the performance of the DiaRD HCV RTqPCR kit, which was designed to quantitate HCV RNA in a broad dynamic range. We showed that this novel HCV RNA quantification kit exhibited good performance with a sensitivity of 98.8%, a specificity of > 99.9%, and an accuracy of 99.6%. Furthermore, correlation analysis performed on 85 HCV RNA positive samples delivered high concordance with the expected HCV RNA values for the DiaRD HCV RTqPCR and the comparator kits, Artus HCV QS-RGQ kit version 1 (QIAGEN GmbH, Germany) and NeuMoDx HCV Quant assay (Qiagen). Moreover, the observed discrepancy >  ± 0.5 log10 (ranging from 0.59 to 1,2 log10) in only a few plasmas supported similarity between the results of the novel kit and the comparator kits. The results of the kit were reproducible with a CV of ≤ 11.73% for intra-assay, ≤ 17.16% for inter-assay, and ≤ 15.38% for inter-load repeats. The novel kit had the ability to give positive results with a clinical sample having HCV RNA load of 20.8 IU/ml with a 95% certainty. Primers and probe used in the novel kit were highly specific, which gave positive results with all major HCV genotypes without any false-positive reaction with other pathogens.

In HCV RNA positive clinical samples, linear regression analysis showed a strong correlation between the HCV RNA levels measured with novel kit and those of the comparator tests. The obtained R^2^ values of 0.915 indicate that more than 91% of the measurements obtained with the novel kit are comparable to those of the comparator kits. These results indicated that the measurement outcomes of the novel DiaRD-HCV kit are highly consistent, and HCV RNA load in clinical samples can be accurately measured. Besides, the slope of the linear regression equation (y = 0.902x + 0395) demonstrates that the measurement values of the DiaRD-HCV kit are nearly like those of the comparators, allowing researchers and clinical practitioners to compare test results. In agreement with our results, previous studies also found high concordance between qPCR methods. A recent study showed that a fully automated microfluidic RT-qPCR system was highly correlated with the Roche cobas AmpliPrep/cobas TaqMan HCV Test, version 2.0 (r2 = 0.949 [14]. Another study reported a correlation (R^2^ = 0.828, p = 0.001) of HCV RNA levels in clinical samples detected by the Artus HCV RT PCR and RTA RT-PCR assays [12]. In a study, quantitation performance of the Abbott Real Time HCV assay and the Roche Cobas TaqMan HCV assay was compared, and it was reported that HCV RNA loads detected with the two assays were highly correlated (*R*^2^ = 0.94) [13]. A recent study performed on 39 HCV RNA positive samples found a high correlation (R^2^ = 0.97) between the Aptima HCV Quant Dx,and Roche Cobas 6800 platform (C6800) systems [9]. These data indicate that even if there are small differences between commercial kits, most of them able to quantitate HCV RNA loads within comparable ranges.

To show the agreement between the quantitative results of DiaRD HCV RTqPCR and comparator kits, Bland–Altman analysis was performed by drawing a scatter plot of the differences against the means of measurements obtained from two tests. Accordingly, while the DiaRD HCV RTqPCR kit assay was measured slightly higher, the mean difference of 0.12 log10 IU/ml was within the acceptable clinical threshold of ± 0.5 log10 IU/mL, with most measurements falling within the 95% confidence interval. Like our result, in a previous study, the Bland–Altman analysis performed on the results of the fully automated microfluidic RT-qPCR system and the Roche cobas AmpliPrep/cobas TaqMan HCV Test, version 2.0 showed that the bias between the two methods were distributed almost evenly in the detection range [14]. In another study, the mean difference between RTA-RT PCR and Artus RG RT-PCR assays in QCMD reference samples was found to be 0.4 log IU/ml [12]. The other study performed to compare diagnostic performance of a new developed one-step real-time PCR assay with Cobas/Taqman HCV Test v2.0 (Roche) found a significant correlation on virus load results of two systems (p = 0.012) [15]. Our findings align with previous studies that reported high concordance between different qPCR systems for HCV RNA quantification.

Before using any qPCR kit for routine diagnosis, it is very important to know the limit of detection/analytical sensitivity of the assay to be sure for its detection ability the low-level viremia to understand those at risk of being mis-diagnosed [16]. According to the manufacturer’s instructions, the limit of detection of the NeuMoDx HCV Quant assay and artus HCV QS RGQ kit version 1 was 8 IU/ml and 21 IU/ml, respectively. A previous study showed that the analytical sensitivity of the HCV specific NASBA assay was 100–150 IU/ml [10]. In another study, the limit of detection of a microfluidic RT-qPCR system was reported as approximately 12 IU/ml [14]. In a recent study, it was reported that a laboratory optimized quantitative RT-PCR method had a limit of detection of 42.6 IU/mL (95% CI, 32.84 to 67.76 IU/mL) [17]. The current study confirmed that The DiaRD HCV RTqPCR kit can detect HCV RNA ≥ 20.8 IU/ml in the samples with a 95% certainty. This finding suggests that the DiaRD HCV kit can reliably detect HCV RNA even in samples with low viral loads, making it suitable for both diagnosis and treatment monitoring.

The reason for the discrepancy between PCR assays may be due to the difference in sample volumes used in RNA extraction: i.e.,200 μl for DiaRD HCV RTqPCR, 500 μl for the artus-HCV RTqPCR systems, and 700 μl for the NeuMoDx system [17]. Also, the elution volume can result in a difference in the RNA quantification. RNA extracted from a high sample volume and resuspended in low-elution buffer increases the detection rate in low-viral-load samples [18]. This argument is supported by one sample not detected in the DiaRD HCV RTqPCR kit, being positive with a 66 IU/ml HCV RNA with the NeuMoDx HCV system. The other reasons may also be the different target size and regions used for detection of viral genome [19]. It was stated that using a short target increased the sensitivity of PCR method [17]. The DiaRD HCV RTqPCR kit targets to amplify the 105-base pairs region within the 5'untranslated region (5'UTR) of the HCV genome, while the Artus HCV QS-RGQ kit uses primers for specific amplification of a 240 bp region of the HCV genome. The other reason might be the difference in analytic sensitivities of the kits [15]. The 95% LoD of the DiaRD HCV RNA RTqPCR assay was 20.8 IU/ml, which is equal to the LoD value (21 IU/ml) of Artus HCV QS-RGQ kit, however it is higher than that of the NeuMoDx HCV system (LoD value of 8 IU/ml). The negative result of the novel kit on the sample having the 66 IU/ml HCV RNA load with the NeuMoDx HCV system might be resulted from its higher LoD value than that of comparator kit. It is most probable this sample might be also negative with the Artus HCV QS-RGQ kit. Unfortunately, we were not able to test this sample with the Artus HCV QS-RGQ kit to clarify this argument. The discrepancy between PCR systems can be related to viral loads in clinical samples. In agreement with this statement, a previous study showed that Roche C6800 system yielded mostly lower viral loads than the Cobas Ampliprep/Cobas TaqMan v2.0 and the Abbott RealTime assay towards to low viral loads, and higher values in the upper viral loads [20]. In our study the difference on the viral loads was not restricted to low or high RNA levels. Of the 12 clinical samples showing differences of > 0.5 log10, six had RNA loads > 4 log10. In the discrepancy samples, the novel kit detected slightly high viral loads in nine samples, of which five had RNA load of > 4 log10. These factors responsible for discrepancy emphasize the importance of assay-specific parameters when interpreting results.

Several studies have shown that HCV genotypes did not significantly affect performance of qPCR kits and did not make a difference in the quantitation performance of the kits [13, 20]. A recent study indicated that quantification of HCV RNA loads by the VERIS HCV assay, and the Abbott RealTime HCV assay showed well correlated for all HCV genotypes, except genotype 4 [21]. Parallel to these results we could not find a significant difference in the quantification of the HCV genotype 1a, 1b, 2, 3a, 5, and 6 in SeraCare’s reference materials. These genotypes could be quantitated with low differences ranging from 0.01 to 0.26 log10 IU/ml. However, in genotype 4 having viral load of 4.16E + 04, a −0.61 log10 difference was observed. Low differences observed in quantification of HCV genotypes between the DiaRD HCV RTqPVR kit and reference values support the robustness of the assay across diverse genotypes. Although we have not been able to detect HCV genotypes in clinical samples to expand accuracy of our results on genotypes circulating in study population, recent studies on the Turkish population have shown that genotype 1 is predominant, accounting up to 95% of HCV strains, followed by genotype 3 [22, 23].

The intra-assay, inter-assay, and inter-load assay reproducibility studies of the novel kit detected standard deviation (SD) values ranging from 6.20 × 10^1^ to 8.54 × 10^3^, and from 2.30 × 10^1^ to 1.43 × 10^4^, and 1.13 × 10^2^ to 5.69 × 10^2^ IU/ml values, respectively. The CV values of this kit were ≤ 11.73% for intra-assay, ≤ 17.16% for inter-assay, and ≤ 15.38% for inter-load repeats. A study using a fully automated microfluidic RT-qPCR system demonstrated that the assay had a reproducibility of SD ≤ 0.16 log10 IU/ml [17]. Another study reported a reproducibility with 2.52% and 1.33% CVs for inter- and intra-assays, respectively for a novel real-time RT-PCR assay [24]. In current study, the mean intra- and inter-assay CV for NeuMoDx HCV Quant Assay was reported as < 5% [8]. According to the instructions of *artus*® HCV RG RT-PCR Kit, CV values for intra-assay and inter-assay were 6.34% and 9.98%, respectively. According to results of six HCV RNA levels tested in three replicates per level in 12 runs on four days by using the AmpliPrep/COBAS® TaqMan® HCV Qualitative Test, v2.0 kit, the CV values were reported between 9 and 54%. It was indicated that CV values ​​below 15% are generally considered acceptable [25]. The coefficient variation values ​​of the DiaRD HCV RTqPCR kit agreed with those of existing commercial kits used in routine diagnosis of HCV infection.

By using results of the comparator kits, the diagnostic sensitivity and specificity of the novel kit were found to be ≥ 99.9%. In agreement with our results, a recent study performed with loop-mediated reverse transcription isothermal amplification demonstrated a sensitivity of 91.5% and specificity of 100% [26]. According to kit’s instruction, the sensitivity and specificity of the COBAS® AmpliPrep/COBAS® TaqMan® HCV Qualitative Test, v2.0 are 100% and 100%, respectively (https://www.aphl.org/programs/infectious_disease/Documents/CAPCTM%20HCV%20v2%20US-IVD%20PI%20rev4%20(Dx%20claim).PDF). These values are 100% and 98.6% for the artus® HCV QS RT-PCR Kit (https://www.qiagen.com/gb/products/diagnostics-and-clinical-research/infectious-disease/artus-hcv-pcr-kits). Both sensitivity and specificity of Abbott RealTime HCV kit are 100% (https://extranet.who.int/prequal/sites/default/files/whopr_files/PQDx_0450-027-00_AbbottRealTimeHCV_PR_v3.0.pdf). DiaRD HCV TRqPCR kit showed good performance in detection and quantification of HCV RNA levels, with similar sensitivity and specificity values as compared to another commercial methods. Detecting all HCV genotypes without any false positive results with the tested pathogens provided additional data regard to accuracy of the novel kit.

Despite its strengths, this study has some limitations. The performance characteristics of the DiaRD kit were not evaluated on a larger cohort of clinical samples with diverse HCV genotypes, nor was it tested for its ability to monitor viral load changes in patients undergoing treatment. Future studies should focus on these aspects to enhance the generalizability of our findings and validate the kit’s performance in a broader clinical context. The other limitation is that due to financial limitation, we could not test all samples being compared across all kits included in the comparison. It’s possible that a sample showing the positive result with NeuMoDX HCV kit but negative with the novel kit might be also negative with Artus HCV QS-RGQ kit, other comparator kit. So, the negative result obtained by the novel kit may not be false negative.

In conclusion, the high correlation between quantitative results of the DiaRD-HCV RNA RTqPCR kit and the comparator kits, along with its high sensitivity, specificity, and accuracy indicates the diagnostic reliability of this novel kit. Besides, its analytical sensitivity supports the ability of the test to detect low viral loads. The DiaRD kit’s ability to detect all major HCV genotypes [1–6] further highlights its clinical utility. These results indicate that the DiaRD HCV RTqPCR kit has the potential to become an integral tool for diagnosis and quantitation of HCV RNA in patients.

## Data Availability

No datasets were generated or analysed during the current study.
